# Genetic diversity and family groups detected in a coyote population with red wolf ancestry on Galveston Island, Texas

**DOI:** 10.1186/s12862-022-02084-9

**Published:** 2022-11-14

**Authors:** Tanner M. Barnes, Melissa Karlin, Bridgett M. vonHoldt, Jennifer R. Adams, Lisette P. Waits, Joseph W. Hinton, Josh Henderson, Kristin E. Brzeski

**Affiliations:** 1 College of Forest Resources and Environmental Science, Michigan Technological University, Houghton, MI USA; 2 Department of Physics and Environmental Science, St. Mary’s University, San Antonio, TX USA; 3 Department of Ecology and Evolutionary Biology, Princeton University, Princeton, NJ USA; 4 Department of Fish and Wildlife Sciences, University of Idaho, Moscow, ID USA; 5 Wolf Conservation Center, South Salem, NY USA; 6 Galveston Island Humane Society, Galveston, TX USA

**Keywords:** Coyote, De-introgression, Genetic introgression, Ghost allele, Hybrid, Red wolf

## Abstract

**Background:**

Hybridization can be a conservation concern if genomic introgression leads to the loss of an endangered species’ unique genome, or when hybrid offspring are sterile or less fit than their parental species. Yet hybridization can also be an adaptive management tool if rare populations are inbred and have reduced genetic variation, and there is the opportunity to enhance genetic variation through hybridization. The red wolf (*Canis rufus*) is a critically endangered wolf endemic to the eastern United States, where all extant red wolves are descended from 14 founders which has led to elevated levels of inbreeding over time. Red wolves were considered extirpated from the wild by 1980, but before they disappeared, they interbred with encroaching coyotes creating a genetically admixed population of canids along coastal Texas and Louisiana. In 2018, a genetic study identified individuals on Galveston Island, Texas with significant amounts of red wolf ancestry. We collected 203 fecal samples from Galveston for a more in-depth analysis of this population to identify the amount of red wolf ancestry present and potential mechanisms that support retention of red wolf ancestry on the landscape.

**Results:**

We identified 24 individual coyotes from Galveston Island and 8 from mainland Texas with greater than 10% red wolf ancestry. Two of those individuals from mainland Texas had greater than 50% red wolf ancestry estimates. Additionally, this population had 5 private alleles that were absent in the North American reference canid populations used in this study, which included 107 southeastern coyotes, 19 captive red wolves, and 38 gray wolves, possibly representing lost red wolf genetic variation. We also identified several individuals on Galveston Island and the mainland of Texas that retained a unique red wolf mitochondrial haplotype present in the red wolf founding population. On Galveston Island, we identified a minimum of four family groups and found coyotes on the island to be highly related, but not genetically depauperate. We did not find clear associations between red wolf ancestry estimates and landscape features, such as open green space or developed areas.

**Conclusion:**

Our results confirm the presence of substantial red wolf ancestry persisting on Galveston Island and adjacent mainland Texas. This population has the potential to benefit future red wolf conservation efforts through novel reproductive techniques and possibly through de-introgression strategies, with the goals of recovering extinct red wolf genetic variation and reducing inbreeding within the species.

**Supplementary Information:**

The online version contains supplementary material available at 10.1186/s12862-022-02084-9.

## Background

Hybridization and the resulting admixture, the movement of alleles from one species into another, occurs naturally in many organisms and is thought to be an important evolutionary process [1–6]. This process may be increasing due to the breakdown of reproductive barriers associated with anthropogenic activities, such as human translocations and habitat fragmentation [7–9]. Hybridization can be harmful to populations if hybrid offspring are sterile or if outbreeding depression occurs [9]. Additionally, admixture leads to a loss of genetic distinctiveness of the parent species which can affect the persistence of rare taxa [10–13]. For instance, hybridization with introduced mallard ducks (*Anas platyrhynchos*) has led to population declines of the New Zealand gray duck (*Anas superciliosa*) [12] and the endemic Hawaiian duck (*Anas wyvilliana*) [9, 14].

Alternatively, hybridization can be a source of genetic rescue to restore fitness in critically endangered populations or species when crossed with populations or subspecies with a more diverse gene pool [15, 16]. For example, introductions of Texas panthers (*Puma concolor*) to Florida assisted in the recovery of the Florida panther (*Puma concolor coryi*) that was experiencing reduced fitness due to fixed deleterious traits from decades of low population size [17–20]. Today, developing technologies such as cloning and genome editing tools are providing new means of genetic rescue [21]. Genome editing and enhanced reproductive techniques may prove useful in supplementing endangered taxa with genetic variation thought to be extinct (ghost alleles) that may only be present in frozen cryo-banks or living hybrid individuals [15, 22, 23]. For instance, genes of the extinct Galapagos tortoise (*Chelonoidis elephantopus*) were detected within admixed individuals of the Wolf Volcano Giant-Tortoise (*Chelonoidis becki*) [24], and these admixed individuals are now being selectively bred to capture the largest genomic representation of the extinct Galapagos tortoise to hopefully recover the species [22].

Whether hybridization is a conservation concern, or an adaptive management tool depends on a variety of factors, such as the size of an endangered population, the current genetic variation present, and importantly the strengths and weaknesses of reproductive barriers operating in a system [2]. For effective conservation planning in the face of contemporary anthropogenic landscape changes, studying the ancestry and population structure of hybrid populations will be critical in understanding the conservation value of admixed individuals.

We investigate ancestry and population structure of admixed coyotes (*Canis latrans*) on Galveston Island, Texas, an island where coyotes with red wolf (*Canis rufus*) ancestry were recently discovered, despite red wolves being declared extinct from the region since 1980 [25]. Endangered red wolves and coyotes interbred when the red wolf suffered severe population declines during the twentieth century and became isolated in Texas and Louisiana [26]. Although, red wolves and coyotes have a complex history of introgression [92], fearful of red wolves’ extinction due to hybridization, disease, and persecution, the United States Fish and Wildlife Service (USFWS) initiated a large trapping effort in the 1970s to create a captive breeding population [27–29]. This effort ended in 1980 and ultimately just 14 individuals from Texas and Louisiana became the founders of all extant red wolves [28].

Although red wolves were considered extinct in Texas and Louisiana, coyotes with large body sizes continued to be reported [30] and recently two studies independently discovered significant red wolf genetic ancestry in coyotes in both Texas and Louisiana [25, 31]. These discoveries suggest that admixed coyotes carrying red wolf genomic ancestry may be common along the Gulf Coast, and that red wolf genetic variation can persist at relatively high levels without human intervention. In North Carolina, studies suggest that despite coyote presence, red wolves more often select conspecific mates or admixed mates rather than coyotes [32, 33]. Finding admixed coyotes with significant amounts of red wolf ancestry along the Gulf Coast, four decades after they were considered extinct in the wild, warrants further investigation into the extent ancestry is present and potential mechanisms that promote the persistence of red wolf genetics in admixed coyotes.

Galveston Island represents an ideal system to better understand mechanisms that promote the persistence of red wolf ancestry given its coyote population likely experiences reduced gene flow with southeastern coyotes on the mainland and it is a relatively closed system. Additionally, Galveston Island’s landscape spans habitats between a rural and urban interface. This provides an opportunity to begin examining patterns of red wolf ancestry distribution, gene flow on and off the island, pack structure, and if habitat selection is a function of canid ancestry. For example, larger carnivores will avoid areas with high human density and modify their behavior as human density increases [34]. Following that red wolf/coyote hybrids in North Carolina are larger than coyotes [35], we hypothesize that coyotes with red wolf ancestry on Galveston may be selecting more undeveloped habitats thereby forcing smaller coyotes into more developed areas.

To better understand the distribution of red wolf ancestry in this population, our objectives were to (1) estimate red wolf ancestry within the population of Galveston Island coyotes, (2) evaluate baseline genetic variation of Galveston Island coyotes and compare it to adjacent mainland Texas coyotes to measure restricted gene flow and inbreeding, (3) estimate relatedness and genetic structure of coyotes on Galveston Island, and (4) describe the distribution of red wolf ancestry within different habitat features. We conducted a systematic noninvasive fecal survey across Galveston Island paired with tissue collection from roadkill, as well as opportunistic sampling from National Wildlife Refuges on mainland Texas from August 2019 to February 2021. This study provides a crucial step in understanding how endangered red wolf ancestry is distributed on the landscape and the mechanisms that reinforce the persistence of red wolf alleles.

## Results

### Sample collection

**Fig. 1:**
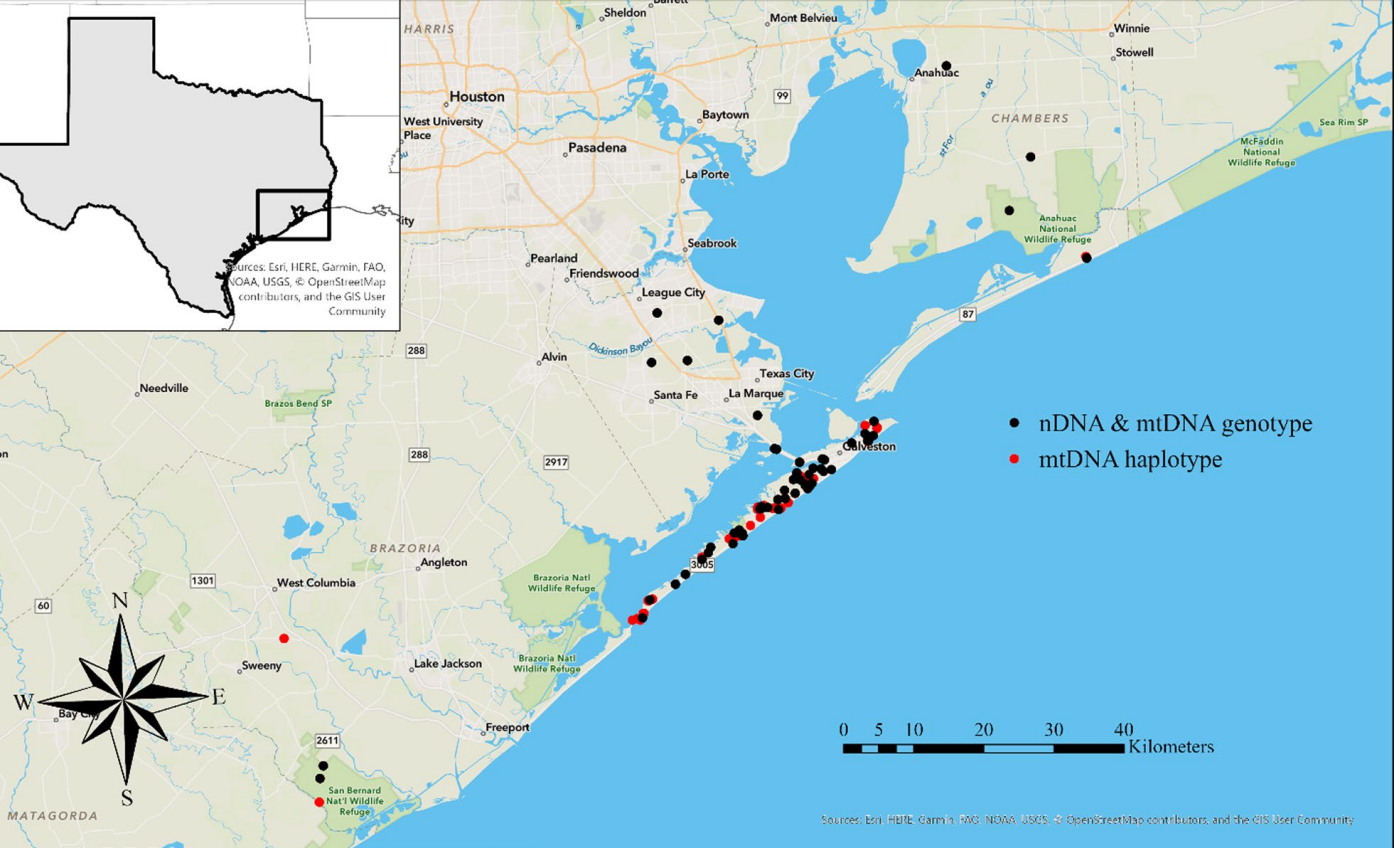
Collection locations of all DNA samples we successfully sequenced. Black dots represent samples with both mitochondrial DNA (mtDNA) haplotypes and nuclear DNA (nDNA) genotypes; red dots represent samples that only have mtDNA haplotypes

We collected a total of 229 fecal samples and 32 tissue samples from southeastern Texas. A total of 168 fecal samples were collected systematically along 25 transects across Galveston Island, Texas, an additional 61 fecal samples were collected opportunistically during a pilot study on Galveston Island in August 2019 and on mainland Texas throughout the duration of the study. Twenty-one of the tissue samples were collected from Galveston Island and 11 tissues were collected from mainland Texas throughout the study (Fig. 1).

### Mitochondrial DNA results

**Fig. 2:**
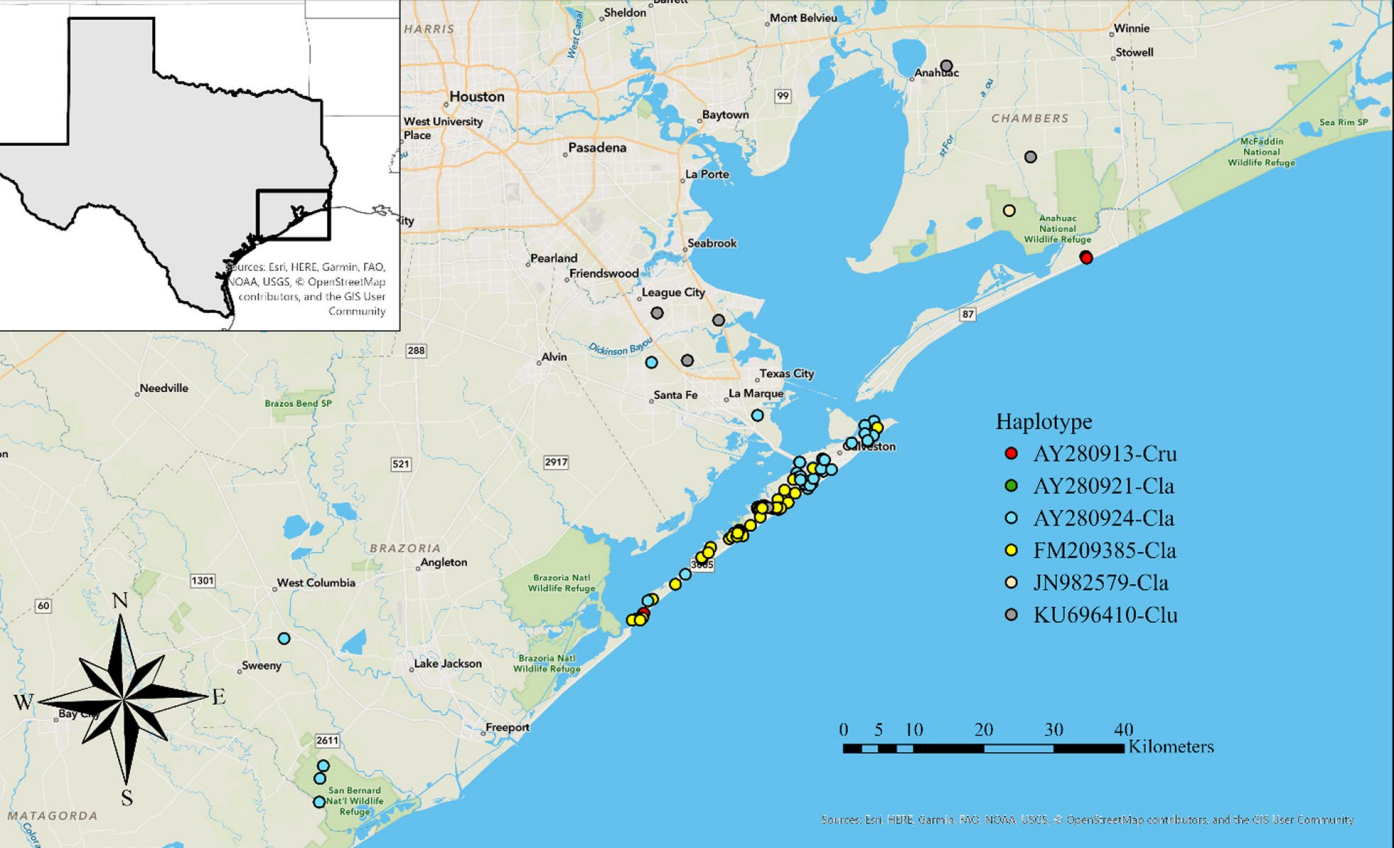
Mitochondrial DNA haplotypes from coyote fecal and tissue samples detected on and surrounding Galveston Island, Texas. Haplotype accession numbers from NCBI GenBank, species code Cru represents (*Canis rufus*), code Cla (*Canis latrans*), code Clu (*Canis lupus*)

We extracted DNA from 222 fecal samples and 32 tissue samples for genetic analysis. We sequenced an approximately 200 base pair segment of the mitochondrial DNA (mtDNA) from the cytochrome B control region to confirm the matrilineal species assignment of each sample. We successfully obtained mtDNA haplotypes at the cytochrome B control region for 94 fecal samples, for a 42% success rate of mtDNA amplification, and for all 32 tissue samples. Based on species identification from mtDNA haplotypes, we removed 9 samples that were not wild canids: 8 domestic dogs and 1 otter. We identified four mitochondrial haplotypes on Galveston Island that matched haplotypes previously published on NCBI GenBank (AY280924, FM209385, KU696410, and AY280913) (Fig. 2).

Two samples from Galveston Island matched the only haplotype found in extant red wolves (AY280913; [36]), one sample matched a haplotype that was most associated with gray wolves (KU696410), and the rest were represented by known coyote haplotypes (AY280924 and FM209385). Of the mainland Texas samples, we identified the same four haplotypes found on Galveston and an additional three that matched or clustered with coyote haplotypes (see mtDNA analysis below; AY280913, JN982579, and a new haplotype OM392562 not previously published). Three samples from the mainland matched the red wolf haplotype and five matched the same gray wolf haplotype found on Galveston (Additional file 2: Fig. S1)*.*

### Microsatellite DNA (nDNA) results

We genotyped samples at 15 microsatellite loci for individual identification, estimates of red wolf ancestry, and to assess population structure of coyotes on Galveston Island, Texas. This multi-locus microsatellite panel has been used extensively in the past for identifying red wolf X coyote hybrids [31, 36–39]. Fecal samples were genotyped multiple times (4–6 replicates) to ensure accuracy, and we successfully generated consensus genotypes for 61 fecal samples, for a 34% fecal genotyping success rate. We successfully genotyped all 32 tissue samples collected on Galveston Island and mainland Texas. We did not obtain a nDNA genotype from 33 fecal samples for which we successfully sequenced mtDNA haplotypes, where we did not obtain a nDNA genotype for any of the individuals with a red wolf mtDNA haplotype. The highest PID_SIBS_ for five loci was 0.0081, thus any combination of five loci would ensure our ability to distinguish between individuals and still be below the PID_SIBS_ threshold of 0.01 (1 out of 100 siblings are predicted to have matching genotypes). We performed a matching analysis in GenAlEx and identified a total of 51 individuals from Galveston Island and 17 individuals from mainland Texas. One sample from Galveston Island was later revealed to be a domestic dog and was removed from further analyses, leaving 50 individuals from Galveston Island (see Additional file 1 for genotypes). We confirmed sex from 47 of the 50 samples from Galveston Island (16 females and 31 males). The number of detections per individual ranged from 1 to 4. Three of the tissue samples from Galveston Island matched a fecal sample.

### Analytical results

**Fig. 3:**
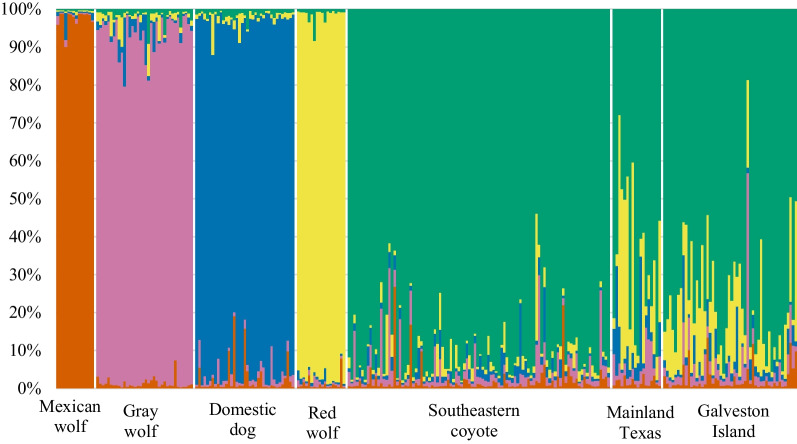
Bar plot of estimated *Canis* nDNA posterior probability ancestry assignments from program STRUCTURE with five genetic clusters (K = 5). Each bar represents ancestry coefficients for one individual. The populations correspond to domestic dogs (*C. lupus familiaris*), gray wolves (*C. lupus*), Mexican wolves (*C. lupus baileyi*), red wolves (*C. rufus*), and southeastern coyotes (*C. latrans*). Our mainland Texas and Galveston Island samples are the last two populations

We determined the amount of red wolf ancestry from nDNA genotype data for 50 Galveston Island coyotes and the 13 Texas mainland coyotes (Fig. 3). Based on posterior probability assignments of ancestry from program STRUCTURE v2.3.4 [40], we determined that Galveston Island coyotes carried an average of 13% (standard deviation 0.11; range (0.9–37%)) red wolf nDNA ancestry (Fig. 3).

**Fig. 4:**
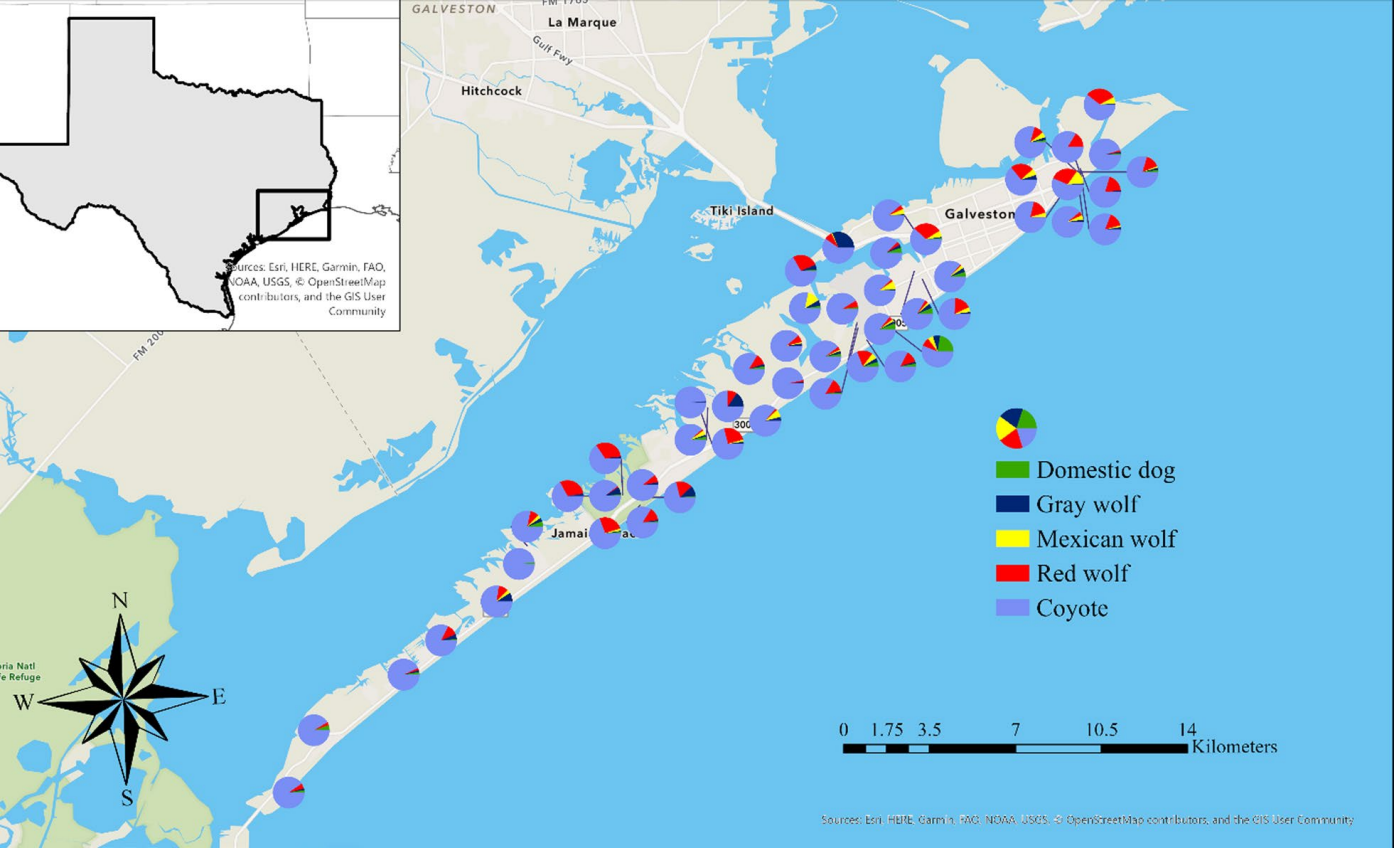
Pie charts representing individual ancestry of coyotes on and near Galveston Island, Texas. Ancestry estimates inferred using the program STRUCTURE K = 5

We found no statistical difference in ancestry between the sexes (one sample t-test, *p* = 0.66). Coyotes from mainland Texas had on average 21% (standard deviation 0.197) red wolf ancestry (range = 2.0–55.8%) (Fig. 4).

**Fig. 5:**
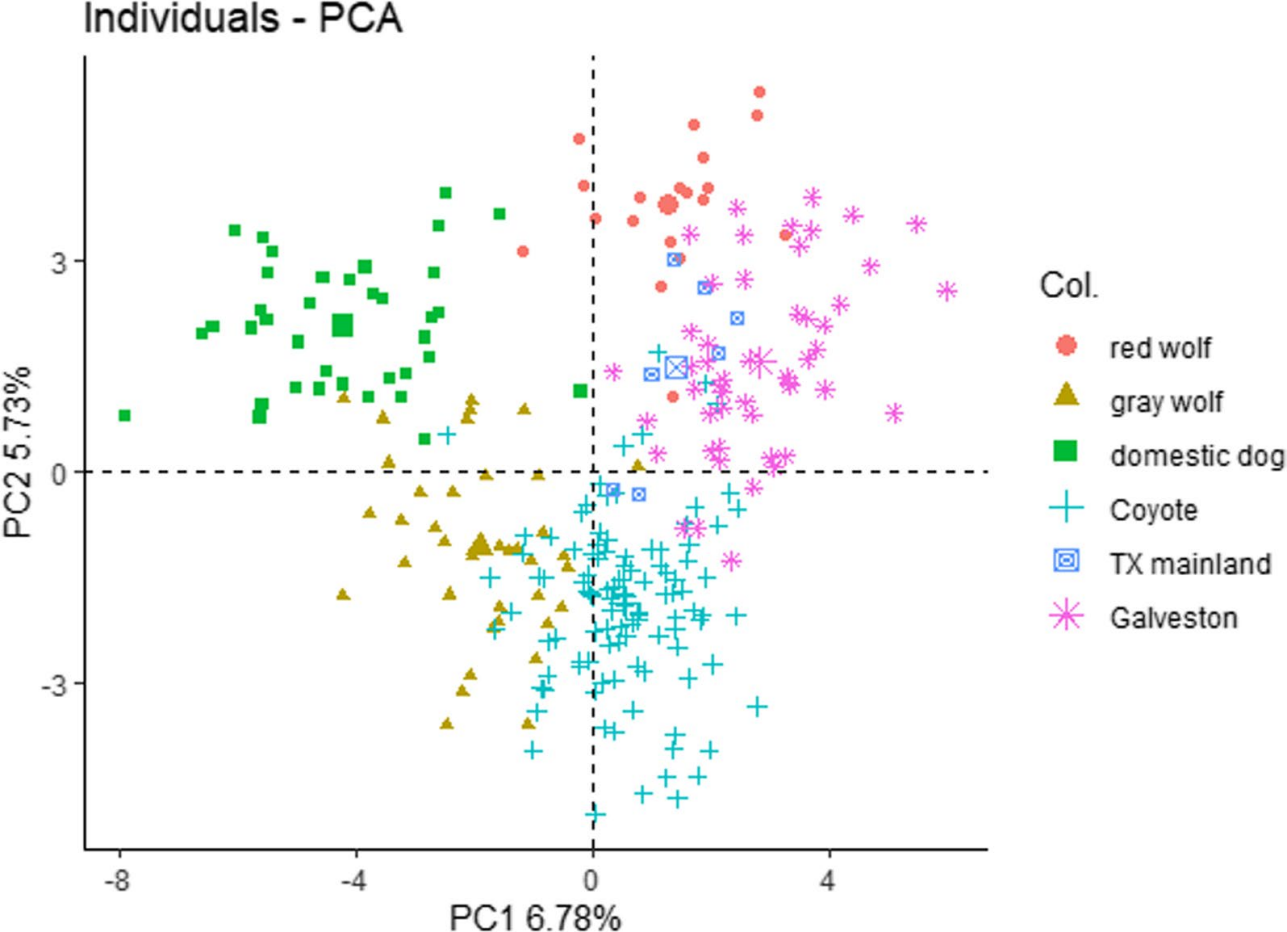
Principal component analysis of sampled *Canis*. Red wolf (*Canis rufus*) reference group are red circles, gray wolf (*Canis lupus*) are yellow triangles, reference coyotes (*Canis latrans*) are green squares, Texas mainland samples from this study are blue cross, and Galveston Island, Texas samples from this study are purple shapes

We used a principal components analysis (PCA) and found that clusters were consistent with taxonomic classifications and previous analyses [25, 41]. Galveston Island and mainland Texas coyotes spanned PC2 between the red wolf and coyote reference groups (Fig. 5). Notably, several Galveston Island and mainland samples cluster very closely to red wolf reference samples.

We compared genetic variation of Galveston Island and Texas mainland coyotes to all our North American reference *Canis* samples. We found the lowest level of genetic differentiation between southeastern reference coyotes and Galveston Island coyotes (F_ST_ = 0.075), followed by Texas mainland samples and reference coyotes (F_ST_ = 0.093) (Table 1).

**Table 1:** Estimated pairwise F_ST_ among reference *Canis* species and sample from this study calculated from 15 loci of nDNA microsatellite data

	Mexican wolf	Gray Wolf	Domestic dog	Red wolf	Southeastern coyote	TX Mainland	Galveston Island
Mexican wolf	–						
Gray wolf	0.278	–					
Domestic dog	0.305	0.110	–				
Red wolf	0.344	0.170	0.182	–			
Southeastern coyote	0.246	0.075	0.111	0.145	–		
TX Mainland	0.291	0.132	0.153	0.149	0.093	–	
Galveston Island	0.296	0.132	0.160	0.152	0.075	0.103	–

Galveston Island (H_O_ = 0.729) and mainland Texas (H_O_ = 0.722) samples also had similar heterozygosity estimates to our reference coyote groups (0.725). We found that Galveston Island and mainland samples had five private alleles compared to reference groups. Galveston Island coyotes showed no loss of heterozygosity or increased inbreeding coefficients (F_IS_ = 0.0151) compared to other groups (Table 2). We further estimated pairwise relatedness for Galveston Island and found that 41 out of 50 Galveston coyotes had at least one parent–offspring or full sibling equivalent relationship assignment (r ≥ 0.50), and 47 out of 50 had a half-sibling equivalent relationship assignment (r ≥ 0.25). Only three individuals were considered unrelated to all other genotyped individuals (r < 0.20).

**Table 2:** Diversity statistics derived from 15 nDNA microsatellite loci for each reference group of *Canis* in North America and samples from on or adjacent to Galveston Island, Texas

Population	n	H_o_	H_e_	A_R_	N_PA_	F	F_IS_
Red wolf	19	0.650	0.638	3.83	1	− 0.050	− 0.0084
Gray wolf	38	0.649	0.746	4.63	0	0.121	0.1436
Domestic dog	38	0.602	0.721	4.65	4	0.142	0.1777
Mexican wolf	14	0.458	0.443	2.43	1	− 1.85E^−4^	0.0042
Southeastern coyote	107	0.725	0.813	5.69	20	0.110	0.1136
TX Mainland	7	0.722	0.722	4.62	2	− 0.005	0.0778
Galveston Island	50	0.729	0.785	4.60	3	0.027	0.0151

*H*_*O*_ observed heterozygosity, *H*_*E*_ expected heterozygosity, *A*_*R*_ allelic richness, *N*_*PA*_ number of private alleles, *F* fixation index, *F*_*IS*_ inbreeding coefficient

**Fig. 6:**
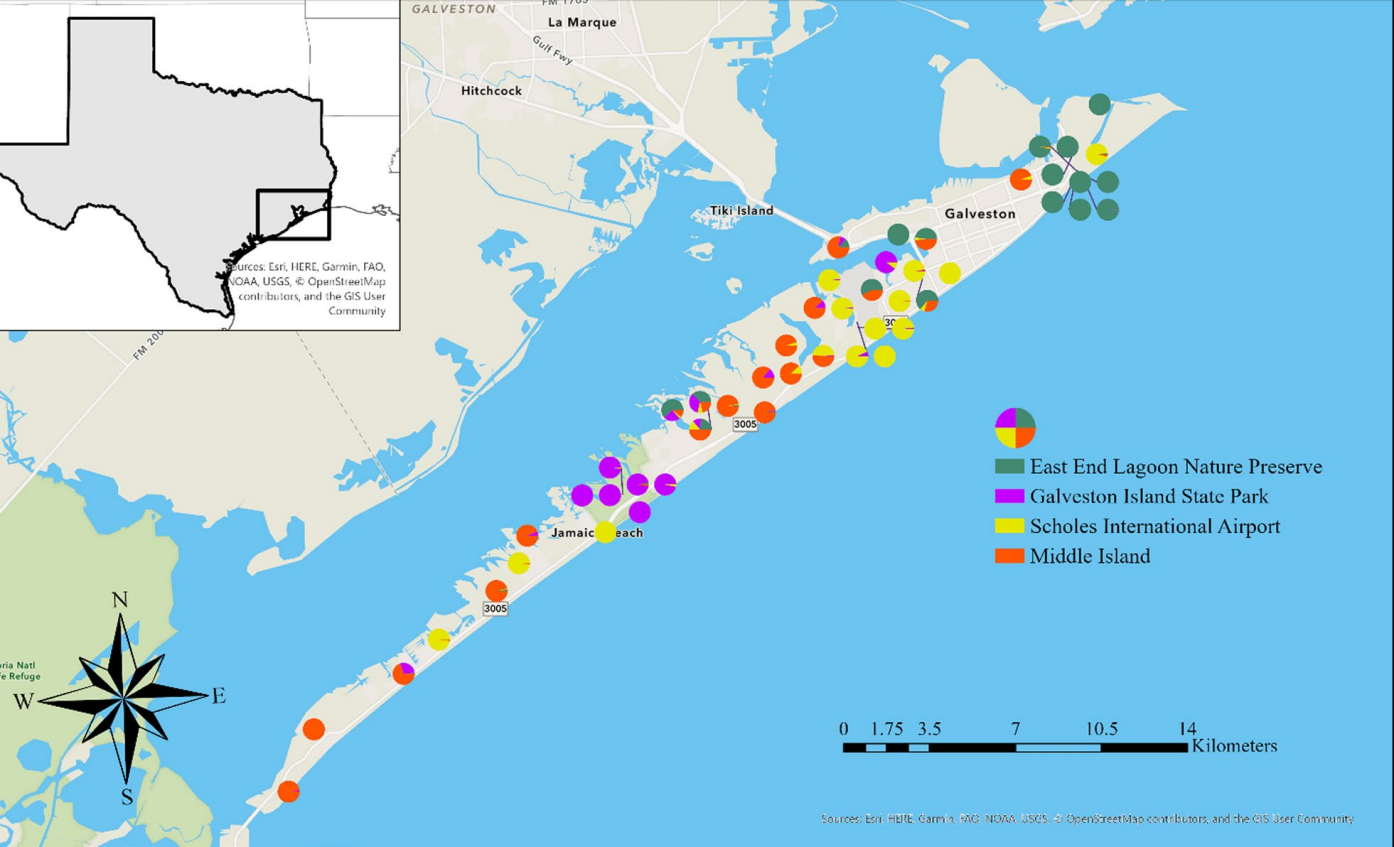
Population genetic substructure (K = 4) from program STRUCTURE of individuals on Galveston Island, Texas, likely representing family groups across the island. Pie charts with multiple colors represent individuals not clearly assigned to one family group

We used Bayesian assignment tests and identified four main family groups spread across the island based on delta K calculated using STRUCTURE HAVESTER v0.6.94 [42]. Forty-two out of 50 individuals we sampled had high assignment to one of the four family groups (Q ≥ 0.8), which we named based on geographic location: East End Lagoon Nature Preserve (n = 10), Scholes International Airport (n = 13), Middle Island (n = 12), and Galveston Island State Park (n = 7; Fig. 6). The remainder eight individuals likely represented unsampled family groups.

We investigated the correlation between red wolf ancestry of family groups and variation in habitat features (e.g., developed, or undeveloped habitat). We found that the family groups of East End Lagoon Nature Preserve and the Galveston Island State Park both overlapped with undeveloped habitat, while the groups from Scholes International Airport and the middle of the island overlapped with developed habitat. We found that undeveloped habitat types were correlated with significantly higher red wolf ancestry proportions than developed habitat (X̄ = 0.155 > 0.0898, one sample t-test, p = 0.023). However, when we grouped all individuals into three category types (open, low intensity developed, high intensity developed), there was no difference in mean red wolf ancestry between habitat types when accounting for family groups (ANOVA, p = 0.99).

## Discussion

We evaluated the ancestry, genetic variation, and genetic structure of the coyote population on Galveston Island, Texas where red wolf ancestry was previously detected in two roadkill individuals [25]. We detected 24 individuals within the population that carried at least 10% red wolf ancestry, and ten individuals with 25% red wolf ancestry. Notably, six of 13 individuals surveyed from the mainland had greater than 30% red wolf ancestry, and two had greater than 50%, which warrants further research to identify the extent of ancestry present and habitats that may be associated with red wolf ancestry on the mainland. We also documented a small proportion of ancestry assigned to other reference species including Mexican wolf, gray wolf, and domestic dog. It is not uncommon to find STRUCTURE analyses with some proportion of ancestry assigned to these other canid groups given the complex evolutionary history of canids in North America as well as common ancestry among some species [43]. In our study, other canid groups (e.g., not red wolf or coyote) do not make up a significant proportion of the samples in this system, and all credible intervals from assignment to other species overlapped with 0, indicating there was not significant evidence to support ancestry from those species.

We further detected the presence of the species-specific red wolf mtDNA haplotype both on and off Galveston Island. This haplotype has only been detected in the wild, outside of the North Carolina red wolf population, by Murphy and colleagues (2019) in southwestern Louisiana, a location that was part of the last remaining stronghold of wild red wolves [44, 45]. Galveston Island coyotes had similar levels of genetic variation as the reference southeastern coyote populations (Table 2), and we did not detect significant amounts of inbreeding despite the presence of related individuals, suggestive of gene flow between Galveston Island and mainland coyotes.

The main mechanism promoting the persistence of red wolf ancestry is not likely isolation due to water between the mainland and the island given the significant amount of red wolf ancestry found on mainland Texas and high genetic diversity of Galveston Island canids. Other mechanisms such as prey selection, territorial aggression, or body size assortative mating like seen in North Carolina [33, 46] may be of importance in this system.

One limitation of this work is we may be underestimating red wolf ancestry in these samples. Our reference red wolf population represent 13 of the 14 genetic founders of the captive breeding population, three offspring of the fourteenth genetic founder, and three additional animals who were selected for the captive breeding population but never reproduced. Although this represents all the genetic diversity remaining in the extant red wolf population, this is a vast underrepresentation of the red wolf genetic diversity prior to the population bottleneck it underwent in the twentieth century. For instance, in the mid-1900s, red wolf/coyote hybrids were documented using morphometrics in Texas [47, 48], Louisiana [49, 50], Arkansas [51], Missouri [52], and Oklahoma [53]. These were the first states coyotes colonized as they migrated east. Given we only compared our samples from Texas to coyotes within these states or east of these states, if early red wolf/coyote hybrids colonized the southeastern states from TX, LA, AK, MO, or OK, we could be underrepresenting red wolf ancestry because historic red wolf genetic variation within these coyotes would appear as southeastern coyote genetic variation, not red wolf. We did detect five private alleles that were not found in any of the reference populations. Although it’s possible these alleles could have been detected in a larger set of reference samples, these alleles could represent unique red wolf genetic variation that was lost when the species experienced the bottleneck to just 14 individuals. Indeed, previous work using genomic methods found potential ghost red wolf alleles in coyotes from this region [25], demonstrating that more work is required to explain the proportion of red wolf ancestry within this population of coyotes.

We found two main mtDNA maternal lineages on Galveston Island. Interestingly, these haplotypes were divided spatially with one lineage in the west side of the island and one in the east side. From these, we identified four main family groups, while some individuals did not assign to any group. These individuals could be migrants or we may have missed family groups due to sampling biases. For instance, there was a 7 km distance on the west side of the island where we could only sample along the main road and the public beach because the rest was private land we could not access. Areas such as this created gaps in our sampling where we could be missing additional family groups. Additionally, two mtDNA haplotypes from fecal samples on the west side of Galveston Island matched the red wolf haplotype, but we were not able to generate genotypes; we could be missing an important family group with high amounts of red wolf ancestry in this area. From the groups we did identify, the ones with the largest percentage of red wolf ancestry were found in The East End Lagoon Nature Preserve and the Galveston Island State Park (Fig. 6). These are well managed natural areas of the island, yet our analysis to determine whether more natural landscapes support red wolf ancestry were inconclusive. When we grouped families into developed and undeveloped areas of the island, we found that undeveloped areas held family groups with significantly higher red wolf ancestry than developed areas. But this pattern was not supported when we grouped individuals by habitat characteristic between high-intensity developed, low-intensity developed, and open habitats on Galveston Island (p-value = 0.54). Sampling biases could be impacting our ability to detect an association between ancestry and landscape features. We collected 26 canids in open habitats, 15 in low-intensity, and 9 in high-intensity developed habitats given it was more difficult to locate scats from wild canids in the City of Galveston than it was in open habitats like the State Park, even though we had reports of canids in both locations.

This research is the first to evaluate Galveston Island coyotes as an entire population, where we are beginning to identify population genetic structure, family groups, and starting to evaluate how habitat may play a role in maintaining high amounts of red wolf ancestry in Texas coyotes. Yet, much more needs to be understood to determine the mechanisms maintaining red wolf ancestry in coyotes, such as whether there is a selective benefit of red wolf/coyote hybridization. Hybridization may have provided an adaptive advantage to expanding coyotes by introducing genetic material already filtered by natural selection in southeastern environments [54]. This process has been documented in northeastern coyotes, where hybridization with eastern wolves (*Canis lupus lycaon*) has resulted in selection for larger body size and skeletal proportions [43, 55]. If a similar adaptive advantage is true in the southeast, coyotes with red wolf ancestry may be more fit in certain environments, promoting persistence of red wolf genetic variation. Multi-generational admixture can produce a diversity of phenotypes and genotypes [56–58]. We have determined that the Galveston Island and adjacent mainland coyote population does have a diversity of genotypes with some individuals having little to no red wolf ancestry and some having 50–60%. Important next steps are to evaluate the diversity of phenotypes within the population and identify regions of admixed genomes that may provide an adaptive advantage from past red wolf introgression.

## Conclusion

We evaluated a population of coyotes with red wolf ancestry along the Gulf Coast of Texas. The study of these admixed coyotes has important implications for red wolves, which are critically endangered and may soon be in need of conservation action. First, admixed populations can act as reservoirs of historic or lost genetic variation [25, 54, 59], or as a source for genetic rescue, which could prove important as contemporary red wolves have become more inbred over time [60]. Through genome editing tools or de-introgression strategies, admixed individuals could be bred to partially recover the extinct species genotype [15, 23]. Second, the USFWS established that a large habitat of 170,000 acres in size is needed to reintroduce red wolves in the wild [61]. Although this criterion is currently under review, this requirement limits the available options for additional red wolf reintroductions. Conditions that maintain red wolf ancestry may be less restrictive than previously thought and areas that support admixed populations may be able to help inform future red wolf reintroduction sites. Third, there is little known about red wolf ecology prior to historic population declines and the red wolf/coyote admixed individuals along the Gulf Coast represent a rare opportunity to learn about the ecological impacts and natural histories of a species thought to be regionally extinct.

## Methods

### Study area

Our study was located south of Houston, Texas on and around Galveston Island, San Bernard Wildlife Refuge, and Anahuac National Wildlife Refuge. This region is humid and subtropical, where hurricanes are common during the summer and fall seasons [62]. Galveston Island is a barrier island situated approximately 80 km south of Houston on the Texas Gulf Coast. It is 160 square kilometers, 43.5 km long, and no more than 4.8 km at its widest point. It has a year-round population of 48,000 residents [63] and supports many tourists throughout the year. Galveston Island is completely disconnected from the mainland, and only has two access bridges, from highway 45 at the North end and San Luis Pass on the west end. The closest distance to the mainland is approximately 1 km at San Luis Pass. San Bernard National Wildlife Refuge is ~ 65 km west of Galveston in East-central Texas. It is a ~ 185 km^2^ wildlife refuge that is a mix of salt marsh, coastal prairies, and bottomland hardwood forests [64]. The Anahuac National Wildlife Refuge is ~ 40 km east of Galveston Island and is a part of the Texas Chenier Plain Refuge Complex which covers 140 km^2^ [65].

### Field methods

We systematically sampled Galveston Island for feces for noninvasive genetic sampling. We collected fecal samples and recorded their GPS location along 25 designated transects distributed east to west (Additional file 1: Fig. S2) across the island during three field seasons, January 2020, July 2020, and January 2021. Each transect was routinely walked or driven over multiple days to retrieve fresh fecal samples. Based on a pilot study in August 2019 to test fecal collection protocols (Additional file 1), we collected fecal samples by swabbing them with a sterile cotton swab that was subsequently placed in a 2 mL tube of Longmire buffer. Additionally, the whole fecal sample was collected in a labelled paper bag and dried for future diet analysis. We also opportunistically collected fecal samples and recorded their GPS location from mainland Texas, including National Wildlife Refuges surrounding Galveston to compare Galveston Island to nearby parts of Texas. Swabs were stored in a freezer at − 20 °C and whole fecal samples were stored in a clean dry area at Michigan Technological University (MTU), Houghton, USA. We collected roadkill tissue samples from Galveston Island and mainland Texas. Coyote tissue was placed on silica or ice and transported to MTU for long term storage at − 20 °C. All research was approved by the MTU Institutional Animal Care and Use Committee (#1438689) and the Texas Parks and Wildlife Department (SPR-0220-020).

### Molecular lab methods

We extracted DNA from fecal samples using a modified QiAmp Fast DNA Stool protocol (Qiagen, Inc., Hilden, Germany), in a laboratory dedicated to low-quality DNA at MTU. We extracted DNA from tissue samples using a DNeasy Blood and Tissue Kit (QIAGEN, Valencia, CA) following the manufacturers protocol in a laboratory dedicated to high-quality DNA samples. We amplified a portion of the cytochrome B region of the mitochondrial genome via polymerase chain reaction (PCR) to confirm DNA isolated from fecal samples was collected from wild coyotes and to gather information on matrilineages, which is useful in differentiating red wolves and coyotes [66]. Extant red wolves are represented by a single mitochondrial haplotype, so we compared the mitochondrial lineages of our generated sequences with previously published sequences to assess if they had the red wolf haplotype.

For low quality DNA extracted from fecal samples, we targeted a ~ 200 base-pair fragment using primers ScatSeqF: 5ʹ-CCATGCATATAAGCATGTACAT-3ʹ and ScatSeqR 5ʹ-AGATGCCAGGTATAGTTCCA-3ʹ [36]. The PCR mix consisted of 10 mM of each primer, 0.2 mM dNTPs, 1 × Buffer II, 2.5 mM MgCl_2_ and 0.5 µL Amplitaq Gold (Applied Biosystems) in a 15 µL reaction with 1.5 µL of DNA extract. We used an Eppendorf Mastercycler Gradient Thermal Cycler (Eppendorf, Hamburg, Germany) using an initial denaturation step of 95 °C for 10 min, 40 cycles (95 °C for 30 s, 48 °C for 45 s, 72 °C for 60 s) and a final extension for 7 min at 72 °C. For high quality DNA extracted from tissue we targeted a ~ 420 base-pair fragment using primers Thr-L: 15,926 5ʹ-CAATTCCCCGGTC TTGTAAACC-3ʹ and DL-H 16,340: 5ʹ-CCTGAAGTAGGAA CCAGATG-3ʹ [67]. The PCR mix consisted of 10 mM of each primer, 0.2 mM dNTPs, 1 × Buffer II, 2.5 mM MgCl_2_ and 0.5 µL Amplitaq Gold (Applied Biosystems) in a 10 µL reaction with 1 µL of DNA extract. The Eppendorf Mastercycler Gradient Thermal Cycler conditions were an initial 10-min denaturation at 95 °C, 40 cycles (95 °C for 30 s, 55 °C for 45 s, 72 °C for 60 s) and a final extension for 7 min at 72 °C. Each PCR was run with a negative control from DNA extractions to monitor for contamination. We visualized each PCR product on a 2% agarose gel run at 115 V for one hour. Samples that contained nonspecific amplification were reamplified with an annealing temperature of 58 °C. We enzymatically cleaned samples using the product ExoSapIT [68] on an Eppendorf Mastercycler Gradient Thermal Cycler following manufacturers protocol and sent samples to GENEWIZ (New Jersey, U.S.A.) for Sanger Sequencing; mitochondrial sequences were viewed, trimmed, and aligned with Geneious software v2021.1.1 [69].

We compared our generated sequences to all previously published sequences in the NCBI BLAST database using Geneious. The samples confirmed to be from the target sequence were aligned using a pairwise MUSCLE alignment in Geneious using 8 iterations. The shorter sequences that were amplified using the Scatseq primers were matched to a longer sequence from the Thr-L 15,926 and DL-H 16,340 primers for haplotype identification. We used a Muscle alignment in Geneious to compare our mtDNA haplotypes to all extant North American canid haplotypes from NCBI GenBank.

We genotyped nuclear DNA (nDNA) at 17 microsatellites at the Laboratory for Ecological, Evolutionary, and Conservation Genetics (University of Idaho, Moscow, U.S.A.) to identify individuals from fecal samples, estimate ancestry, and estimate relatedness. This multi-locus microsatellite panel has been used extensively in the past for identifying red wolf X coyote hybrids [31, 37–39, 66]. We generated genotypes using two multiplexes [37, 38]. The first multiplex contained 0.06 µM of CXX.377, 0.07 µM of CXX. 172, CXX.173, and CXX.250, 0.13 µM of CXX.109, 0.16 µM of CXX.200, 0.20 µM of AHTq121, 0.60 µM of AHT103, 0.71 µM of CXX.20, 1X Qiagen Multiplex PCR Kit Master Mix, 0.5X Q solution, and 1 µL of DNA extract in a 7 µL reaction [31, 39, 70–72]. The second multiplex contained 0.06 µM of FH2010, 0.07 µM of FH2062 and FH2054, 0.10 µM of FH2001, 0.16 µM of FH2145, 0.24 µM of FH2004, 0.36 µM of CXX.225, 0.80 µM of CXX.403, 1X Qiagen Multiplex PCR kit Master Mix, 0.5X Q solution, and 1 µL of DNA extract in a 7 µL reaction [39, 70, 71]. We amplified tissue samples in duplicate and performed up to four and six replicate PCRs for the tissue and fecal samples respectively. We visualized PCR products using a 3130xl DNA Sequencer and scored allele sizes using Genemapper 5.0 (Applied Biosystems, Inc., Foster City, U.S.A.). Assessment of sample quality and genotype screening methods followed those described by Adams and Waits (2007). We calculated Hardy–Weinberg Equilibrium using package Pegas in R v4.0.2 [73] and used a Bonferroni correction that corrects for multiple, simultaneous comparisons [74]. Two loci deviated from Hardy–Weinberg Equilibrium and were removed from downstream analyses. We calculated probability of identity (PID) and probability of identity for siblings (PID_SIBS_) using nine loci in Multiplex 1 with GenAlEx v6.5 [75] and performed a matching analysis in GenAlEx to determine how many individuals were detected in the fecal genotypes after Multiplex 1. Only five loci were necessary to differentiate between individuals, so only unique individuals were amplified with Multiplex 2 (Additional file 1: Fig. S3).

### Analytical methods

Our first objective was to estimate red wolf ancestry proportions in each coyote by assessing the mitochondrial lineage and determining the distribution of red wolf ancestry in nDNA across the island and adjacent mainland Texas. To accomplish this, we estimated gene trees with mtDNA haplotypes using Bayesian methods implemented in BEAST v.1.10.4 [76], with a constant size coalescent tree prior, an uncorrelated lognormal relaxed molecular clock, and a random starting tree. We conducted Markov Chain Monte Carlo (MCMC) analyses with 20 million steps, sampling every 2000 steps, and combined tree estimates from each run with LogCombiner v.1.10.4 [77] with a 10% burn-in. We calculated the maximum clade credibility in TreeAnnotator v.1.10.4 [78] and uploaded the most likely tree in the Interactive Tree of Life v3.6.3 online webtool to visualize the gene tree and mtDNA lineages [79].

To determine red wolf nDNA ancestry, we used a Bayesian assignment method [38] implemented in program STRUCTURE [40]. Galveston Island and mainland samples were assigned ancestry proportions to our *Canis* references. References included Mexican wolves (n = 14), domestic dogs (n = 38), gray wolves (n = 38), red wolves (n = 19) representing 13 of the 14 genetic founders plus descendants of the fourteenth founder of the captive breeding population, and southeastern coyotes (n = 107; Additional file 1: Fig. S4). For the STRUCTURE analysis we set the number of populations (*K*) a priori to five (e.g., Mexican wolf, domestic dog, gray wolf, red wolf, and coyote) and ran 10 independent runs of the admixture model with correlated allele frequencies with a burn-in of 20,000 Markov chain Monte Carlo iterations followed by 50,000 iterations to estimate a posterior probability of ancestry (q; Additional file 1: Table S1). To prevent bias that can arise from including related individuals among our samples, we used the PopFlag prior that uses a Boolean variable to indicate learning samples. Because the Popflag prior can be sensitive to admixed individuals in the reference populations, after an initial STRUCTURE run, we removed reference individuals with ≥ 25% ancestry assignment to a different reference group (i.e., gray wolf with 25% dog ancestry; Additional file 1: Fig. S5). Our final references included Mexican wolves (n = 14), domestic dogs (n = 37), gray wolves (n = 36), red wolves (n = 18), and southeastern coyotes (n = 98). We used a t-test to compare mean red wolf ancestry proportions between males and females and further evaluated genetic clustering with a principal component analysis (PCA) using package Adegenet [80] and package Factoextra [81] in R v4.0.2. We conducted two PCAs, the first with all reference populations (Additional file 1: Fig. S6) and second with a subset (gray wolf, red wolf, coyote, our samples) to better visualize clustering with only one outgroup.

Our second objective was to evaluate the baseline genetic variation of Galveston Island coyotes and compare it to Texas mainland coyotes surrounding the island to measure for restricted gene flow and inbreeding. We calculated standard measures of genetic variation in several ways. First, we calculated observed and expected heterozygosity, which are measures of genetic diversity within a population using program GenALEx [75]. We calculated this metric for all Galveston Island coyotes, Texas mainland samples that were in close geographic proximity to the island, and all *Canis* reference populations (Table 1). Next, we assessed genetic differentiation of Galveston Island, and our Texas mainland samples in comparison to reference populations by estimating pairwise F_ST_ [82] and F_IS_ values per population using package Hierfstat in program R [83]. We additionally calculated allelic richness and private alleles using package PopGenReport in program R [84].

Our third objective was to estimate relatedness, genetic substructure, and describe the distribution of red wolf ancestry on Galveston Island. To identify possible family groups, we first estimated genetic relatedness of Galveston Island coyotes by calculating pairwise relatedness between all individuals on Galveston Island using the maximum likelihood approach implemented by the program ML-Related [85]. We estimated average relatedness of all individuals on Galveston Island and evaluated differences in relatedness between the sexes. Next, we used STRUCTURE to evaluate the genetic substructure of coyotes on Galveston Island. We conducted 10 independent runs for each K value with the admixture model K = 1–10, using 50,000 repetitions after a burn-in of 20,000 Markov chain Monte Carlo iterations. The most likely number of genetic clusters represented by the data was estimated by considering delta K [86], calculated with STRUCTURE HAVESTER v0.6.94 [42]. However, delta K cannot provide support for K = 1 or K = 10 as it is based on the rate of change in log-likelihood between successive K values, so we also used the log-likelihood (Ln Probability) values inferred from STRUCTURE [40]. We used pie charts plotted spatially in ArcGIS Pro to visualize differences in genetic clusters (K = 2–7). We considered individuals to have high assignment to a given inferred cluster if the ancestry proportion (q) was greater than or equal to 0.8. We separated individuals into family groups based on their assignment.

To address our fourth objective, we then separated the family groups by the habitat characteristics based on the GPS location they were collected within (developed or undeveloped), to test for differences in mean red wolf ancestry proportions between developed and undeveloped natural areas of Galveston Island. We used The National Oceanic and Atmospheric Administration (NOAA) geospatial classification system with a 30-m resolution [87]. We ran a F-test to test for unequal variances and then ran a one sample t-test on the ancestry proportions of the family groups within each habitat characteristic. Next, we used NOAA land cover class [87] in ArcGIS Pro and grouped all individuals into three habitat categories: open, low intensity developed, and high intensity developed based on where they were sampled. The open category included habitats with little to no human presence including Galveston Island State Park, East End Nature Preserve, Artist Boat Coastal Heritage Preserve, and Galveston Bay Foundation Conservation Preserve; the low intensity category included green spaces with some development including golf courses, airports, and RV parks; the high intensity category included Galveston City. We conducted an ANOVA in R Studio to test for a difference in mean red wolf ancestry between the three habitat categories using family groups as a random variable.

## Supplementary Information

**Additional file 1.** Microsatellite genotypes, ancestry statistics, and identification of private alleles.

**Additional file 2: Fig S1.** Mitochondrial DNA haplotype gene tree of the cytochrome B region of the mitochondrial control region. Accession numbers are from NCBI GenBank; accession numbers that match sequences from this study are labeled with their detection location. Species code Vv represents the outgroup red fox (*Vulpes vulpes*), Clu is gray wolf (*Canis lupus*), Cll is eastern wolf (*Canis lupus lycaon*), Cla is coyote (*Canis latrans*), and Cru red is red wolf (*Canis rufus*). **Fig S2.** Systematic sample design on Galveston Island, Texas. We noninvasively collected scat samples across 25 transects during three field seasons. **Fig S3.** Probability of Identity for locus combinations. **Fig S4.** Distribution by state of the coyote reference samples used in this study. **Fig S5.** STRUCTURE output including every reference samples. **Fig S6.** PCA with all reference samples. **Table S1.** STRUCTURE q percentage and credible interval of all samples using 15 microsatellites.

## Data Availability

The datasets analyzed during the study are included in this published article and its Additional information files. DNA Sequences deposited to NCBI GenBank accession #OM392562 https://www.ncbi.nlm.nih.gov/nuccore/OM392562.1?report=GenBank.
