# Supplementary material for: Genetic diversity and family groups detected in a coyote population with red wolf ancestry on Galveston Island, Texas
Source: BMC Ecol Evol. 2022 Nov 14;22:134. doi: 10.1186/s12862-022-02084-9 (PMC9664737; doi:10.1186/s12862-022-02084-9)
Supplement: Supplementary file 2 — Additional file 2: Fig S1. Mitochondrial DNA haplotype gene tree of the cytochrome B region of the mitochondrial control region. Accession numbers are from NCBI GenBank; accession numbers that match sequences from this study are labeled with their detection location. Species code Vv represents the outgroup red fox (Vulpes vulpes), Clu is gray wolf (Canis lupus), Cll is eastern wolf (Canis lupus lycaon), Cla is coyote (Canis latrans), and Cru red is red wolf (Canis rufus). Fig S2. Systematic sample design on Galveston Island, Texas. We noninvasively collected scat samples across 25 transects during three field seasons. Fig S3. Probability of Identity for locus combinations. Fig S4. Distribution by state of the coyote reference samples used in this study. Fig S5. STRUCTURE output including every reference samples. Fig S6. PCA with all reference samples. Table S1. STRUCTURE q percentage and credible interval of all samples using 15 microsatellites. [file 12862_2022_2084_MOESM2_ESM.docx]

Supplemental File

Appendix A

DNA from scat can be affected by climatic conditions and time since defecation (Ando et al. 2020). Therefore, prior to our sampling, we performed a pilot study to test the efficacy of different scat collection methods (Rutledge et al. 2009; Miles et al. 2015). Scats were collected in three different ways: swabbing with a cotton swab and place in Longmire buffer, wiping the exterior surface with toothpicks and dried with silica, and collecting whole scats place on ice or dried. All methods provide approximately the same quality sequences. However, swabbing with a cotton swab was the most efficient and cost-effective approach. We also collected whole scats for use in future analyses.

Ando H, Mukai H, Komura T, Dewi T, Ando M, Isagi Y. Methodological trends and perspectives of animal dietary studies by noninvasive fecal DNA metabarcoding. Environmental DNA. 2020 Oct;2(4):391-406.

Miles KA, Holtz MN, Lounsberry ZT, Sacks BN. A paired comparison of scat‐collecting versus scat‐swabbing methods for noninvasive recovery of mesocarnivore DNA from an arid environment. Wildlife Society Bulletin. 2015 Dec;39(4):797-803.

Rutledge LY, Holloway JJ, Patterson BR, White BN. An improved field method to obtain DNA for individual identification from wolf scat. The Journal of Wildlife Management. 2009 Nov;73(8):1430-5.

Appendix B


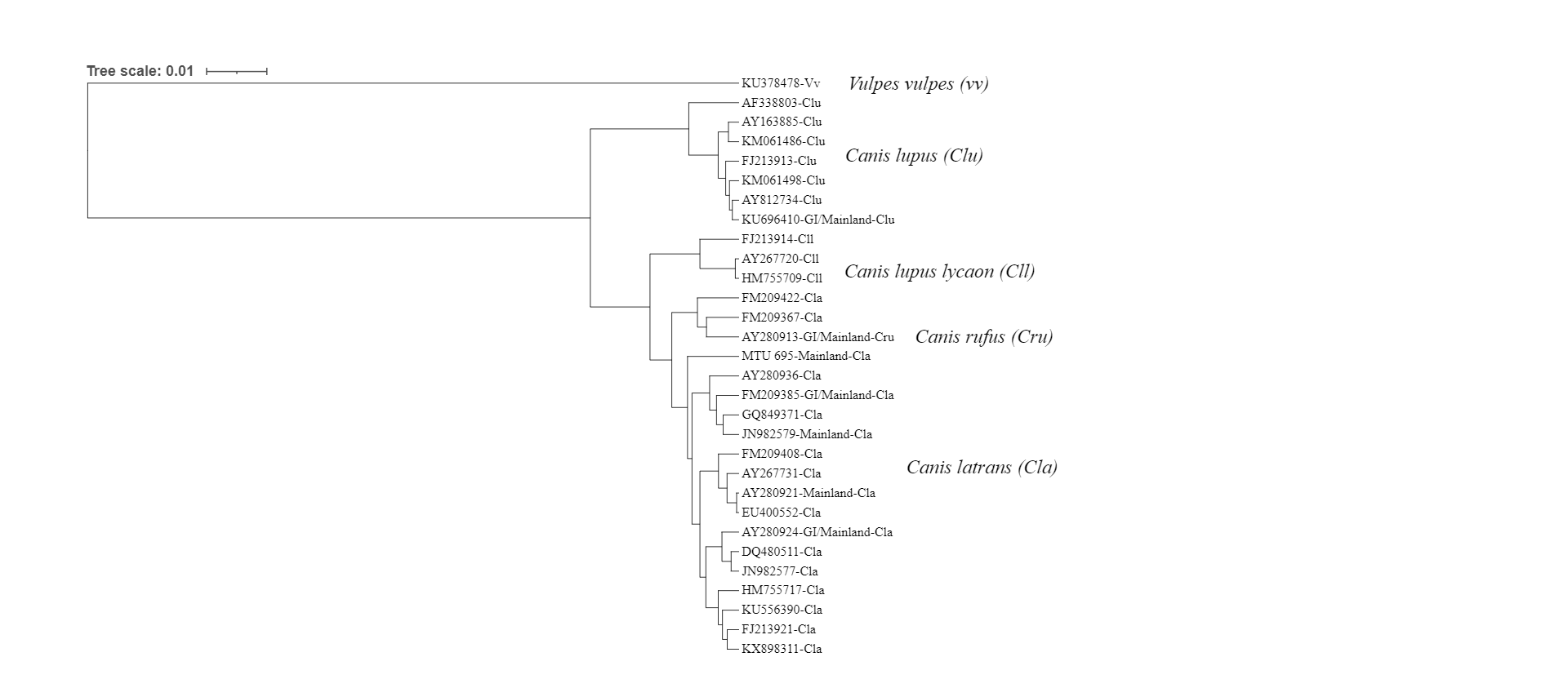


0.9988

0.9999

0.9766

0.9801

0.9472

0.7803

0.9902

0.5903

Supplemental Figure 1. Mitochondrial DNA haplotype gene tree of the cytochrome B region of the mitochondrial control region. Accession numbers are from NCBI GenBank; accession numbers that match sequences from this study are labeled with their detection location. Species code Vv represents the outgroup red fox (*Vulpes vulpes*), Clu is gray wolf (*Canis lupus*), Cll is eastern wolf (*Canis lupus lycaon*), Cla is coyote (*Canis latrans*), and Cru red is red wolf (*Canis rufus*).


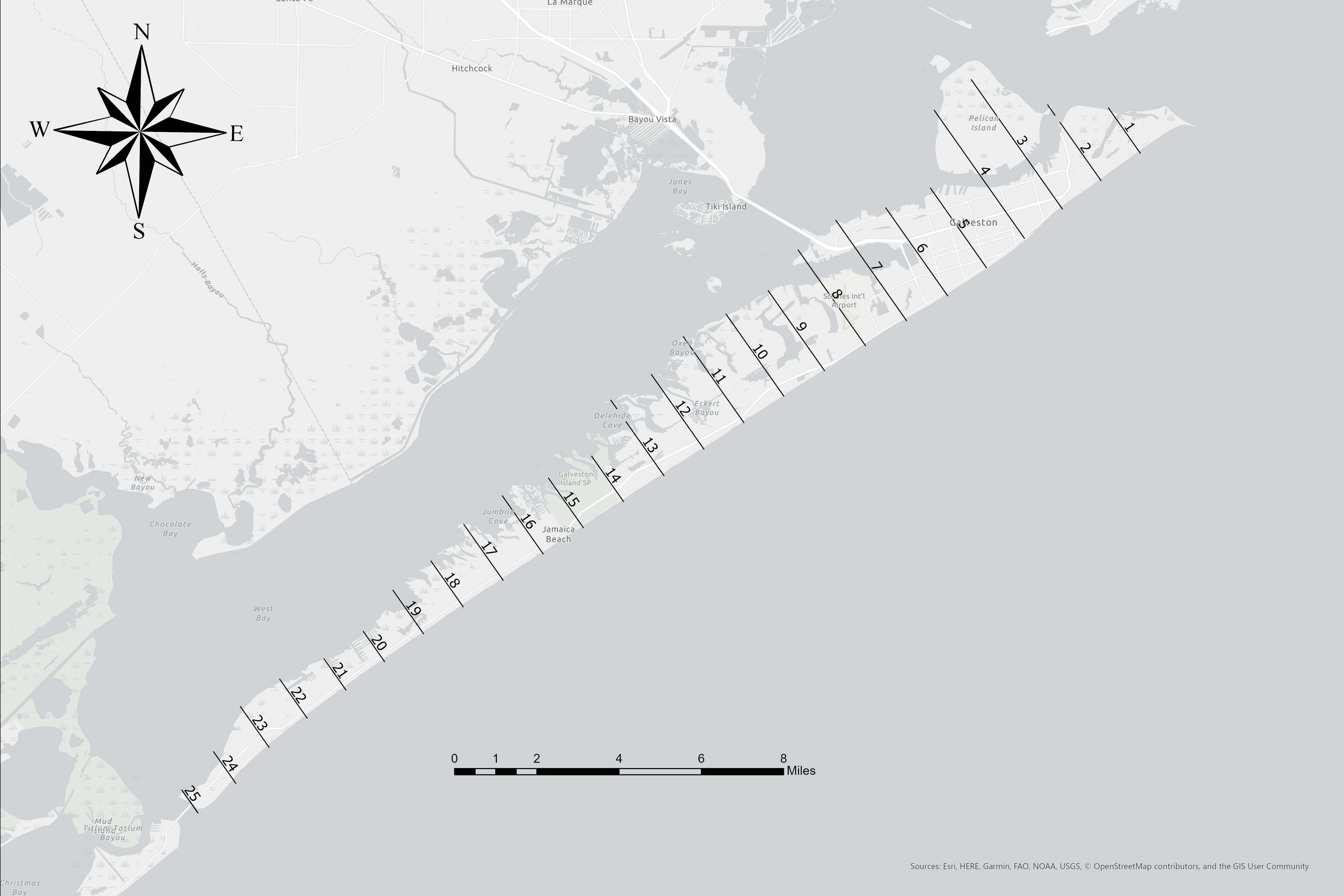


Supplemental Figure 2. Systematic sample design on Galveston Island, Texas. We noninvasively collected scat samples across 25 transects during three field seasons.

Supplemental Figure 3. Probability of Identity for locus combinations.

Supplemental Figure 4. Distribution by state of the coyote reference samples used in this study.

Supplemental Figure 5. STRUCTURE output including every reference samples.


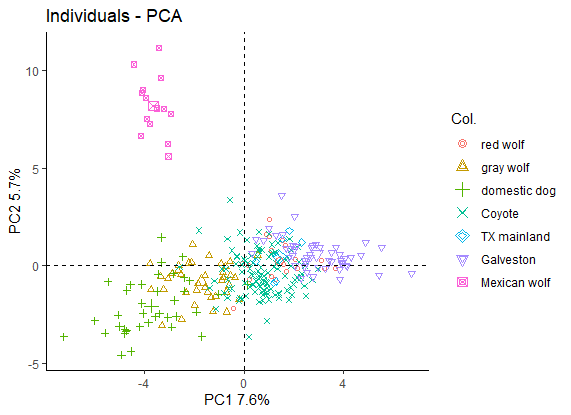


Supplemental Figure 6. PCA with all reference samples.

Supplemental Table 1. STRUCTURE q percentage and credible interval of all samples using 15 microsatellites.

|  | Structure q (%) | | | | | Structure (credible interval) | | | | |
| --- | --- | --- | --- | --- | --- | --- | --- | --- | --- | --- |
| Sample | MW | GW | DG | RW | CY | MW | GW | DG | RW | CY |
| 001 | 3.3% | 2.0% | 2.2% | 7.5% | 85.1% | 0,0.202 | 0,0.134 | 0,0.148 | 0,0.370 | 0.537,1.0 |
| 007 | 1.3% | 1.6% | 1.8% | 13.6% | 81.7% | 0,0.089 | 0,0.105 | 0,0.125 | 0,0.345 | 0.575,1.0 |
| 015 | 0.8% | 6.6% | 8.3% | 2.9% | 81.4% | 0,0.052 | 0,0.313 | 0,0.371 | 0,0.177 | 0.472,1.0 |
| 023 | 0.3% | 0.8% | 1.8% | 21.7% | 75.4% | 0,0.018 | 0,0.048 | 0,0.115 | 0,0.494 | 0.462,0.9 |
| 027 | 0.9% | 0.6% | 0.8% | 13.2% | 84.6% | 0,0.060 | 0,0.033 | 0,0.047 | 0,0.385 | 0.587,1.0 |
| 031 | 2.2% | 28.9% | 1.2% | 3.1% | 64.6% | 0,0.148 | 0,0.628 | 0,0.077 | 0,0.205 | 0.283,0.9 |
| 040 | 0.9% | 0.8% | 2.0% | 5.9% | 90.4% | 0,0.056 | 0,0.051 | 0,0.137 | 0,0.306 | 0.638,1.0 |
| 049 | 2.3% | 9.7% | 4.3% | 55.8% | 27.9% | 0,0.145 | 0,0.338 | 0,0.265 | 3,0.810 | 0.000,0.5 |
| 289 | 1.1% | 1.7% | 2.1% | 19.8% | 75.3% | 0,0.075 | 0,0.107 | 0,0.139 | 0,0.522 | 0.423,1.0 |
| 293 | 0.8% | 0.8% | 2.1% | 22.8% | 73.5% | 0,0.057 | 0,0.048 | 0,0.138 | 0,0.574 | 0.375,1.0 |
| 297 | 6.3% | 17.7% | 11.2% | 8.7% | 56.3% | 0,0.289 | 0,0.717 | 0,0.513 | 0,0.420 | 0.000,0.9 |
| 307 | 0.8% | 2.1% | 5.3% | 35.0% | 56.8% | 0,0.052 | 0,0.147 | 0,0.330 | 5,0.606 | 0.193,0.8 |
| 313 | 10.1% | 0.9% | 8.6% | 3.7% | 76.6% | 0,0.356 | 0,0.056 | 0,0.437 | 0,0.216 | 0.387,1.0 |
| 316 | 0.4% | 0.9% | 2.8% | 34.8% | 61.1% | 0,0.022 | 0,0.058 | 0,0.172 | 7,0.606 | 0.327,0.8 |
| 320 | 0.8% | 7.6% | 5.7% | 3.1% | 82.8% | 0,0.057 | 0,0.417 | 0,0.357 | 0,0.208 | 0.398,1.0 |
| 332 | 0.6% | 0.9% | 1.3% | 25.7% | 71.4% | 0,0.041 | 0,0.053 | 0,0.087 | 0,0.521 | 0.438,0.9 |
| 342 | 1.3% | 3.5% | 2.2% | 14.2% | 78.8% | 0,0.091 | 0,0.238 | 0,0.155 | 0,0.455 | 0.467,1.0 |
| 352 | 0.4% | 1.3% | 1.7% | 34.5% | 62.2% | 0,0.024 | 0,0.080 | 0,0.114 | 6,0.614 | 0.334,0.9 |
| 356 | 1.1% | 1.3% | 8.0% | 10.0% | 79.6% | 0,0.074 | 0,0.086 | 0,0.420 | 0,0.364 | 0.427,1.0 |
| 486 | 13.4% | 7.2% | 3.0% | 22.1% | 54.2% | 0,0.392 | 0,0.391 | 0,0.204 | 0,0.494 | 0.203,0.8 |
| 500 | 3.7% | 7.5% | 3.5% | 8.1% | 77.1% | 0,0.215 | 0,0.369 | 0,0.200 | 0,0.300 | 0.415,1.0 |
| 504 | 1.3% | 0.8% | 1.4% | 30.2% | 66.3% | 0,0.089 | 0,0.049 | 0,0.093 | 0,0.584 | 0.379,0.9 |
| 510 | 0.6% | 0.9% | 3.5% | 47.6% | 47.5% | 0,0.038 | 0,0.057 | 0,0.232 | 4,0.749 | 0.182,0.8 |
| 554 | 2.7% | 7.8% | 5.3% | 4.4% | 79.7% | 0,0.160 | 0,0.411 | 0,0.315 | 0,0.253 | 0.432,1.0 |
| 555 | 2.2% | 1.6% | 2.7% | 2.5% | 91.0% | 0,0.146 | 0,0.112 | 0,0.187 | 0,0.172 | 0.654,1.0 |
| 556 | 3.5% | 1.3% | 3.1% | 41.9% | 50.2% | 0,0.209 | 0,0.083 | 0,0.191 | 4,0.711 | 0.206,0.8 |
| 557 | 0.7% | 1.1% | 1.6% | 8.0% | 88.6% | 0,0.043 | 0,0.065 | 0,0.104 | 0,0.411 | 0.543,1.0 |
| 558 | 0.7% | 0.7% | 1.2% | 1.3% | 96.1% | 0,0.045 | 0,0.046 | 0,0.080 | 0,0.082 | 0.812,1.0 |
| 559 | 0.8% | 8.6% | 1.8% | 44.7% | 44.1% | 0,0.055 | 0,0.404 | 0,0.118 | 0,0.728 | 0.134,0.7 |
| 560 | 1.0% | 1.8% | 2.1% | 10.7% | 84.4% | 0,0.065 | 0,0.121 | 0,0.147 | 0,0.355 | 0.552,1.0 |
| 562 | 2.5% | 4.2% | 2.7% | 50.3% | 40.4% | 0,0.159 | 0,0.248 | 0,0.167 | 1,0.769 | 0.071,0.7 |
| 672 | 0.9% | 3.6% | 2.1% | 9.3% | 84.0% | 0,0.060 | 0,0.203 | 0,0.127 | 0,0.319 | 0.565,1.0 |
| 673 | 0.8% | 1.6% | 6.3% | 21.2% | 70.1% | 0,0.053 | 0,0.109 | 0,0.365 | 0,0.487 | 0.326,0.9 |
| 693 | 1.1% | 3.4% | 1.0% | 5.9% | 88.5% | 0,0.077 | 0,0.207 | 0,0.068 | 0,0.307 | 0.623,1.0 |
| 695 | 0.3% | 4.7% | 1.7% | 2.0% | 91.3% | 0,0.020 | 0,0.244 | 0,0.115 | 0,0.140 | 0.680,1.0 |
| 696 | 1.2% | 30.2% | 3.4% | 4.7% | 60.5% | 0,0.085 | 0,0.660 | 0,0.219 | 0,0.268 | 0.218,0.9 |
| 697 | 0.6% | 2.1% | 1.3% | 19.1% | 76.9% | 0,0.038 | 0,0.143 | 0,0.085 | 0,0.461 | 0.477,1.0 |
| 698 | 3.2% | 2.0% | 1.9% | 26.4% | 66.6% | 0,0.188 | 0,0.134 | 0,0.129 | 0,0.560 | 0.357,0.9 |
| 700 | 3.9% | 1.3% | 1.4% | 34.5% | 59.0% | 0,0.213 | 0,0.084 | 0,0.092 | 0,0.583 | 0.337,0.8 |
| 702 | 0.8% | 3.2% | 15.3% | 2.4% | 78.4% | 0,0.052 | 0,0.198 | 0,0.452 | 0,0.161 | 0.490,1.0 |
| 703 | 1.0% | 17.5% | 3.1% | 7.8% | 70.5% | 0,0.071 | 0,0.671 | 0,0.214 | 0,0.351 | 0.120,1.0 |
| 704 | 1.0% | 0.9% | 1.2% | 29.9% | 67.1% | 0,0.068 | 0,0.052 | 0,0.079 | 0,0.586 | 0.379,0.9 |
| 705 | 1.2% | 0.9% | 9.8% | 9.2% | 78.9% | 0,0.083 | 0,0.057 | 0,0.487 | 0,0.399 | 0.359,1.0 |
| 709 | 2.0% | 0.9% | 3.6% | 7.6% | 85.9% | 0,0.135 | 0,0.059 | 0,0.237 | 0,0.390 | 0.507,1.0 |
| 735 | 5.2% | 1.4% | 51.6% | 23.1% | 18.7% | 0,0.279 | 0,0.089 | 0,0.956 | 0,0.568 | 0.000,0.7 |
| 743 | 1.2% | 5.5% | 1.0% | 3.2% | 89.2% | 0,0.083 | 0,0.247 | 0,0.058 | 0,0.224 | 0.618,1.0 |
| 759 | 11.8% | 3.5% | 4.1% | 1.2% | 79.4% | 0,0.389 | 0,0.190 | 0,0.214 | 0,0.076 | 0.465,1.0 |
| 833 | 0.8% | 2.5% | 2.5% | 3.3% | 90.8% | 0,0.051 | 0,0.173 | 0,0.177 | 0,0.224 | 0.632,1.0 |
| 853 | 0.4% | 1.3% | 2.6% | 8.6% | 87.1% | 0,0.024 | 0,0.080 | 0,0.174 | 0,0.344 | 0.578,1.0 |
| 857 | 0.7% | 1.3% | 1.2% | 36.1% | 60.6% | 0,0.047 | 0,0.078 | 0,0.083 | 3,0.674 | 0.280,0.9 |
| 879 | 0.6% | 3.0% | 1.4% | 6.8% | 88.3% | 0,0.038 | 0,0.156 | 0,0.087 | 0,0.373 | 0.560,1.0 |
| 887 | 2.0% | 0.8% | 1.3% | 1.8% | 94.0% | 0,0.134 | 0,0.051 | 0,0.087 | 0,0.125 | 0.749,1.0 |
| 893 | 0.8% | 2.4% | 1.1% | 10.8% | 84.9% | 0,0.054 | 0,0.165 | 0,0.069 | 0,0.384 | 0.546,1.0 |
| 901 | 0.5% | 1.1% | 1.4% | 1.4% | 95.6% | 0,0.027 | 0,0.071 | 0,0.091 | 0,0.095 | 0.792,1.0 |
| 917 | 1.5% | 3.4% | 2.7% | 3.3% | 89.1% | 0,0.100 | 0,0.176 | 0,0.183 | 0,0.224 | 0.613,1.0 |
| 919 | 0.7% | 4.0% | 1.2% | 1.2% | 92.9% | 0,0.048 | 0,0.255 | 0,0.075 | 0,0.076 | 0.687,1.0 |
| 931 | 1.8% | 0.8% | 5.1% | 6.4% | 85.8% | 0,0.124 | 0,0.050 | 0,0.284 | 0,0.286 | 0.563,1.0 |
| 932 | 0.7% | 10.6% | 12.3% | 6.3% | 70.1% | 0,0.047 | 0,0.354 | 0,0.448 | 0,0.267 | 0.324,0.9 |
| 933 | 2.1% | 2.4% | 10.4% | 6.8% | 78.3% | 0,0.136 | 0,0.171 | 0,0.454 | 0,0.338 | 0.432,1.0 |
| 934 | 3.4% | 3.8% | 1.9% | 24.7% | 66.2% | 0,0.204 | 0,0.227 | 0,0.125 | 0,0.596 | 0.301,0.9 |
| 952 | 1.1% | 0.6% | 0.5% | 6.1% | 91.7% | 0,0.068 | 0,0.035 | 0,0.032 | 0,0.331 | 0.639,1.0 |
| 968 | 0.5% | 1.4% | 1.8% | 0.9% | 95.4% | 0,0.034 | 0,0.092 | 0,0.120 | 0,0.057 | 0.780,1.0 |
| 992 | 0.7% | 9.0% | 7.8% | 26.7% | 55.8% | 0,0.041 | 0,0.428 | 0,0.411 | 0,0.603 | 0.162,0.9 |
| 996 | 0.6% | 0.6% | 1.3% | 14.4% | 83.1% | 0,0.035 | 0,0.036 | 0,0.089 | 0,0.442 | 0.522,1.0 |
| 997 | 3.8% | 6.8% | 5.2% | 4.2% | 80.0% | 0,0.209 | 0,0.374 | 0,0.311 | 0,0.253 | 0.452,1.0 |
| 998 | 12.0% | 0.7% | 10.1% | 27.6% | 49.5% | 0,0.368 | 0,0.045 | 0,0.441 | 0,0.588 | 0.150,0.8 |
| 999 | 5.3% | 5.4% | 6.0% | 1.4% | 81.9% | 0,0.249 | 0,0.326 | 0,0.350 | 0,0.091 | 0.472,1.0 |
| 1000 | 9.8% | 1.3% | 1.3% | 37.0% | 50.6% | 0,0.366 | 0,0.084 | 0,0.088 | 0,0.694 | 0.115,0.8 |
